# Cyanidin-3-O-glucoside (C3G): A natural small-molecule compound for alleviating envenomation symptoms Induced by *Bungarus multicinctus*

**DOI:** 10.1371/journal.pntd.0014207

**Published:** 2026-04-07

**Authors:** Ziyan Zhang, Ningjing Jiang, Manqi Xiao, Shaocong Hu, Qiuju Jia, Deguo Dong, Ming Liao

**Affiliations:** 1 School of Basic Medical Sciences, Guangxi Medical University, Nanning, PR China; 2 Life Science Institute Guangxi Medical University, Nanning, PR China; 3 Air Force Specialty Medical Center, Beijing, PR China; Universidade Federal do Amazonas, BRAZIL

## Abstract

Bungarus multicinctus (many-banded krait) ranks among the world’s most medically significant venomous snakes. Its venom, predominantly composed of α-bungarotoxin neurotoxins in a complex mixture, induces life-threatening respiratory paralysis, pulmonary failure, and often multi-organ dysfunction following envenomation. Building upon our discovery that the chemical dye Cy7-SE attenuates the toxicity of *Bungarus multicinctus* venom, this study employed network pharmacology to analyze molecular docking parameters between Cy7-SE and α-bungarotoxin. We subsequently applied computational virtual screening to identify natural small molecules alleviating symptoms of *B. multicinctus* envenomation, followed by comprehensive in vitro and in vivo validation. Molecular docking revealed that Cy7-SE forms a stable complex with α-bungarotoxin through five hydrogen bonds, exhibiting a binding energy of -8. 49 kcal/mol. Using optimized GridBox parameters derived from this interaction, we performed batch molecular docking against the ZINC database, identifying 3, 118, 296 potential α-bungarotoxin-binding molecules. Through sequential filtering—binding energy ≤ -7 kcal/mol, ADMET prediction analysis, Lipinski’s rule screening, weighted refinement via Pandas library analysis, and final prioritization using PyMOL visualization—coupled with literature mining, the natural compound cyanidin-3-glucoside (C3G) was identified as a promising therapeutic candidate. Molecular dynamics simulations confirmed the stable binding of C3G to α-bungarotoxin. Surface plasmon resonance demonstrated that C3G and α-bungarotoxin have a strong binding affinity. In vivo studies showed that co-injection of high-dose C3G (300-fold molar equivalent to venom) with *B. multicinctus* venom significantly enhanced murine survival rates. Moreover, immediate post-envenomation administration of C3G at this dosage improved 24-hour survival and alleviated histopathological damage in diaphragmatic and pulmonary tissues. Notably, the protective effect of C3G relies on an extremely high molar excess and is mainly limited to co-administration or immediate post-envenomation intervention; this compound acts as a symptomatic ameliorating agent to delay disease progression and mitigate secondary tissue damage, rather than exerting direct or clinically significant venom neutralization, distinguishing it from antivenom.

## Author summary

Bungarus multicinctus envenomation causes life-threatening neurotoxicity and multi-organ failure due to its dominant toxin α-bungarotoxin. Building on our prior discovery that the dye Cy7-SE attenuates the toxicity of this venom, we employed computational strategies (network pharmacology and molecular docking) to identify natural small-molecule compounds that alleviate symptoms of *B. multicinctus* envenomation. We found that Cy7-SE stably binds α-bungarotoxin via five hydrogen bonds (-8. 49 kcal/mol binding energy). Using this interaction model, we virtually screened over 3 million compounds from the ZINC database and identified cyanidin-3-glucoside (C3G) as a top candidate through rigorous binding energy filtering, ADMET analysis, and literature validation. Molecular dynamics confirmed C3G’s stable binding to the toxin, validated by SDS-PAGE affinity assays. Crucially, in vivo studies demonstrated that high-dose C3G (300-fold molar excess to venom) co-injected with *B. multicinctus* venom significantly improved murine survival rates. Immediate post-envenomation C3G administration further enhanced 24-hour survival and mitigated histopathological damage in diaphragm and lung tissues. This study provides the first preliminary evidence that C3G—a naturally occurring flavonoid—exerts symptomatic amelioration effects against krait envenomation, serving as an adjuvant intervention to alleviate toxin-induced tissue injury and delay disease progression.

## Introduction

Snakebite is a severe neglected tropical disease (NTD) that affects 2. 5 million people each year, resulting in the deaths of 81, 000–138, 000 individuals, including rural villagers, agricultural workers, and children [1,2]. As one of China’s top ten venomous snakes, *Bungarus multicinctus* (many-banded krait) produces venom dominated by neurotoxins (3-FTx) [3]. Envenomation by this species primarily manifests as neurotoxic poisoning, with research confirming α-bungarotoxin (α-BGT) as a critical postsynaptic neurotoxin [4–7]. This toxin competitively inhibits acetylcholine receptors (AChRs) at nerve terminals, forming non-functional α-neurotoxin-nAChR complexes that prevent acetylcholine binding. Consequently, neuromuscular transmission is blocked, leading to muscle paralysis, systemic paresis, respiratory failure [8–11], and high mortality without prompt intervention [12].

Current clinical management relies predominantly on antivenom [13], which has significant limitations. Efficacy requires administration before tissue binding of venom components [14], Studies report that even when administered within 4 hours post-envenomation at adequate doses, antivenom fails to reverse neurotoxic effects or prevent respiratory failure [15]; Additionally, antivenom carries risks of allergic reactions (palpitations, rashes, dyspnea), potentially progressing to anaphylactic shock or death [16,17]. Clinically, *B. multicinctus* envenomation often necessitates 5–30 days of mechanical ventilation, increasing risks of complications like ventilator-associated pneumonia and imposing substantial economic burdens [18]. Thus, developing novel adjuvant therapeutics is imperative for reducing mortality and disability.

In recent years, computational drug screening has gained momentum in pharmaceutical development. This approach simulates target-compound interactions [19,20], using molecular docking and dynamics to identify bioactive molecules from large databases [21], offering cost-efficiency and high throughput [22]. It has enabled drug discovery and repositioning [23,24], exemplified by studies on wedelolactone (GPD1 activator against bladder cancer) and lead compound optimization [25,26]. As a cornerstone of virtual screening, molecular docking has successfully advanced anti-infective and anticancer research: elucidating galangin’s insulin resistance mechanisms [27], designing PBP2a inhibitors [28], and developing polyphyllin B as a GPX4-targeting anticancer agent [29]. Notably, Nature published work by David Baker’s team demonstrating de novo design of artificial proteins that neutralize three-finger toxins (3FTxs) in vitro and protect mice against lethal neurotoxicity [30].

Building on our discovery that the chemical dye Cy7-SE attenuates the toxicity of *B. multicinctus* venom, this study employed network pharmacology to analyze molecular docking parameters between Cy7-SE and α-bungarotoxin. We subsequently applied computational virtual screening to identify natural small molecules alleviating symptoms of *B. multicinctus* envenomation, followed by comprehensive in vitro and in vivo validation.

## Methods and materials

### Ethics statement

Animal experiments were approved by the Experimental Animal Ethics Committee of Guangxi Medical University(Approval No. 202507001).

### Experimental animals and husbandry environment

One hundred and seven Kunming (KM) mice (18–22 g, half male and half female) were purchased from Guangxi Medical University Laboratory Animal Center (Guangxi, China, approval No. SYXK【Gui】2025–0005). The mice were kept in standard SPF grade laboratories (temperature 20˚C-25˚C,

relative humidity 50%-65%, free access to water) and fed ordinary feed three times a day for one week. Animal experiments were conducted in accordance with internationally recognized standards for the care and use of laboratory animals. All procedures involving animals were reviewed and specifically approved by the Experimental Animal Welfare and Ethics Committee of Guangxi Medical University (Approval No. 202507001).

### Main experimental reagents and materials

Bungarus multicinctus venom lyophilized powder was obtained from the Snake Venom Research Instituteof Guangxi Medical University and stored at -80˚C. Ultrapure water was filtered by Milli-Q(USA) and then used. CY7-SE was purchased from MedChemExpress (MCE, USA). Sodium pentobarbital was acquired from Sigma-Aldrich Co. LLC (USA). Hematoxylin-eosin (H&E) staining kits were obtained from Solarbio Science & Technology Co., Ltd. (China). C3G was purchased from Shanghai Perfemiker Co., Ltd (China).

### Database and software

PubChem, ZINC, and Pandas databases; AutoDock4, PyMOL, Open Babel, Python 3. 11, ADMET-AI, GROMACS, and SOBTOP software.

### Effect of Cy7-SE mixed with *Bungarus multicinctus* venom on murine survival rate

Lyophilized *B. multicinctus* venom (10 mg) was dissolved in 1 mL PBS to prepare a stock solution, while Cy7-SE (5 mg) was dissolved in 200 μL PBS to generate its stock solution. KM mice were randomly divided into seven groups (n = 10 per group): control (PBS), 2LD50 venom group (0. 09 mg/kg *B. multicinctus* venom), 2LD50 venom + Cy7-SE group (0. 09 mg/kg venom + 0. 9 mg/kg Cy7-SE), 4LD50 venom group (0. 18 mg/kg venom), 4LD50 venom + Cy7-SE group (0. 18 mg/kg venom + 1. 8 mg/kg Cy7-SE), 6LD50 venom group (0. 27 mg/kg venom), and 6LD50 venom + Cy7-SE group (0. 27 mg/kg venom + 2. 7 mg/kg Cy7-SE). Each mouse received a 50 μL intramuscular injection of the designated solution into the hind leg. Survival rates were recorded at 1h, 3h, 6h, 12h, and 24h post-injection.

### Molecular docking analysis of Cy7-SE with α-Bungarotoxin (α-BGT)

Cy7-SE and α-BGT (PDB: 2BTX) structure files were retrieved from the Protein Data Bank. The α-BGT PDB file was imported into AutoDock, where hydrogen atoms were added and water molecules removed before exporting as a PDBQT receptor file. The Cy7-SE PDB file was hydrogenated, designated as the ligand, and rotatable bonds were identified before PDBQT export. A Grid Box enveloping α-BGT was generated and saved as a GPF file for AutoGrid execution. Docking parameters were configured as follows: semi-flexible docking mode (Set Rigid Filename) with α-BGT as receptor and Cy7-SE as ligand; Genetic Algorithm selected with 50 GA runs, 3, 000, 000 maximum evaluations, and 30, 000 maximum generations. After saving parameters, AutoDock was executed. Output files were converted to PDB format via OpenBabel. PyMOL visualized hydrogen bonding interactions and structural context between Cy7-SE and α-BGT. The conformation with lowest binding energy was prioritized, and GridBox parameters were extracted.

### Batch molecular docking analysis of α-BGT with small-molecule compounds

The Tranches subset of the ZINC database was queried using the filters: 3D conformers, In-Stock availability, Reference standard status, charge states (-1/0/ + 1), and lead-like properties. Molecules with LogP < 0 were excluded. The resulting 3D structural archive was downloaded and processed with OpenBabel to split into individual SDF files, which were converted to PDBQT format with hydrogen addition and water removal. The α-BGT PDB file was loaded into AutoDock, and Grid Box parameters derived from the Cy7-SE/α-BGT docking complex were applied. In the Docking Output module, Vina Config (config. txt) was selected. After verifying receptor and ligand assignments, the number of docking modes was set to 10 with other parameters at default. The Autodock Vina configuration file (conf. txt) was validated for consistency within the local working directory. All PDBQT files (small molecules, α-BGT, Cy7-SE), along with conf. txt, were consolidated in one directory. A batch processing script for Autodock Vina (Supporting information) was executed via results. bat, generating binding energy data tables for successful α-BGT-ligand complexes.

### Python-based screening of α-BGT-binding molecules

Aggregated docking results were sorted by ascending binding energy. A Python script (Supporting information) selected the top 5% scoring molecules, followed by extraction of compounds with binding energy ≤ -7 kcal/mol. These molecules were cataloged in a dedicated data table, with corresponding structural files archived for downstream analysis.

### ADMET and lipinski rule screening

The filtered molecules underwent ADMET prediction using the open-source ADMET-AI toolkit. Python environment setup included ADMET Model installation (pip install admet-ai). A custom Python script (Supporting information) performed the analysis, yielding an “admet_filtered. xlsx” file containing ADMET profiles and Lipinski’s rule compliance.

### Screening of α-BGT-binding small molecules via pandas and PyMOL

The Pandas library performed weighted analysis on the results, retaining the top 20% of candidates (script code in Supporting information) to generate the “top_candidates. csv” file. Corresponding structural files of small molecules docked with *B. multicinctus* venom proteins were extracted to a new directory. A Python script invoked PyMOL for batch visualization of ADMET-filtered molecules, extracting the lowest-energy conformation. Protein structures were loaded to render interaction images, while hydrogen bonding interactions between ligands and the α-bungarotoxin receptor were quantified and archived (script in Supporting information. Hydrogen bond data was tabulated, with the top 15 results manually inspected alongside literature evidence to select validation candidates.

### Molecular dynamics simulation of C3G-α-BGT binding

C3G and α-neurotoxin PDB structures were converted to Mol2 format using OpenBabel. SOBTop processed the C3G Mol2 file to generate Gro format output. GAFF force field parameters were assigned to derive topology (top) and include (itp) files, subsequently integrated with protein parameters. Molecular dynamics simulations were conducted using the GROMACS software(script in Supporting information).

### Surface plasmon resonance analysis of C3G and α-BGT

First, the biotinylated α-BGT ligand was stably immobilized on the surface of an SA sensor chip via the biotin-streptavidin system to ensure ligand orientation and high-density capture. Subsequently, in the Kinetics/Affinity program, a series of C3G analyte solutions with a concentration gradient (0μM, 12μM (with replicates), 16μM, 20μM, 24μM, 28μM) were sequentially flowed over the chip surface. The sensorgram formed by the real-time binding and dissociation process between C3G and the immobilized α-BGT was monitored and recorded. To eliminate non-specific signals caused by the solvent, four solvent correction cycles were also included in the experiment. Finally, using the dedicated analysis software of the SPR instrument, the sensorgrams were processed with double referencing and fitted to a 1:1 binding model. This allowed for the calculation of the association rate constant (ka), the dissociation rate constant (kd), and subsequently the equilibrium dissociation constant (KD), thereby quantitatively characterizing the interaction affinity between α-BGT and C3G.

### Effect of C3G mixed with *Bungarus multicinctus* venom on murine survival rate

Healthy KM mice were randomized into five groups (n = 10 per group): control (PBS), venom group (0. 09 mg/kg *B. multicinctus* venom), venom + low-dose C3G group (0. 09 mg/kg venom + 0. 9 mg/kg C3G), venom + medium-dose C3G group (0. 09 mg/kg venom + 1. 8 mg/kg C3G), and venom + high-dose C3G group (0. 09 mg/kg venom + 2. 4 mg/kg C3G). Solutions were vortex-mixed and incubated at 4°C overnight before administering 50 μL intramuscular injections into the thigh muscle. Survival rates were recorded at 1h, 3h, 6h, 12h, and 24h post-injection.

### Impact of C3G on survival and tissue pathology in envenomed mice

Healthy KM mice were randomized into five groups (n = 10): control (PBS injection), venom group (0. 09 mg/kg *B. multicinctus* venom injection), low-dose group (0. 09 mg/kg venom followed immediately by 9 mg/kg C3G at the same site), medium-dose group (0. 09 mg/kg venom followed immediately by 18 mg/kg C3G), and high-dose group (0. 09 mg/kg venom followed immediately by 27 mg/kg C3G). Survival was monitored at 1h, 3h, 6h, 12h, and 24h. At the 6-hour timepoint, diaphragm and lung tissues from the high-dose group were harvested for hematoxylin-eosin (HE) staining and histopathological analysis according to the manufacturer’s protocol.

### Statistical analysis

All experiments were repeated at least three times, and the results were mean ± standard deviation, statistical analysis and graphing were performed using SPSS 20. 0, GraphPad Prism 10. Log-rank test for survival, P < 0. 05 were considered to indicate significantly different.

## Results

### Effect of Cy7-SE mixed with *Bungarus multicinctus* venom on murine survival rate

**Fig 1:**
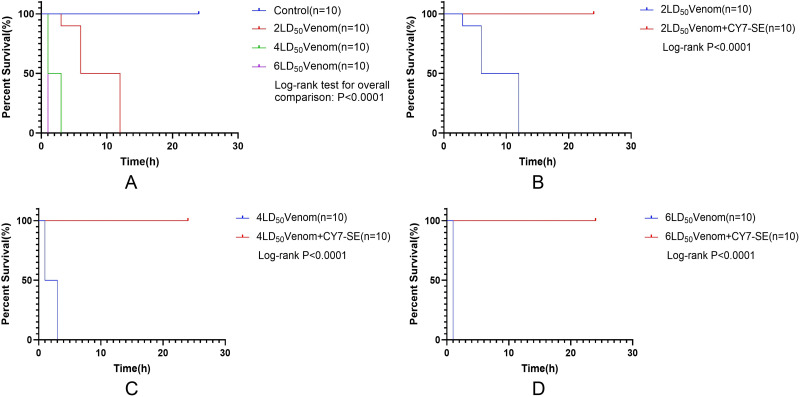
24h survival rates of mice injected with Cy7-SE mixed with varying doses of *Bungarus multicinctus* venom.

As shown in **Fig 1**, compared to the venom-only group, intramuscular co-administration of *B. multicinctus* venom with varying doses of Cy7-SE significantly increased 24-hour survival rates in mice (P <0.0001). This indicates that in vitro mixing of Cy7-SE with the venom attenuates its toxicity to some extent.

### Binding energy, visualization, and GridBox parameters for Cy7-SE and α-Bungarotoxin (α-BGT)

**Fig 2:**
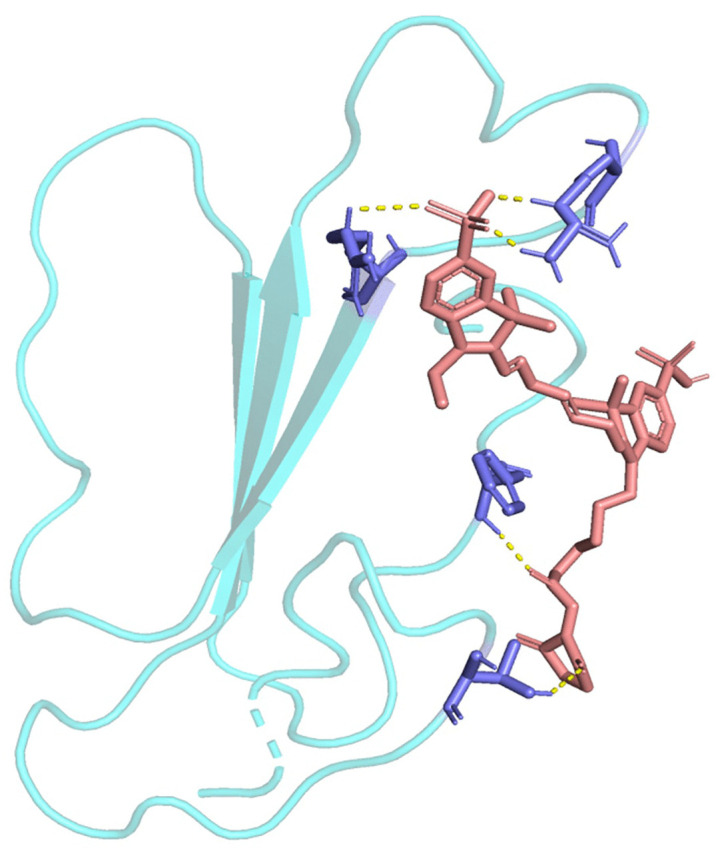
Molecular docking visualization of Cy7-SE with α-bungarotoxin. Cyan: α-bungarotoxin; Blue: Hydrogen-bonded residues; Magenta: Cy7-SE; Yellow dashed lines: Hydrogen bonds.

Molecular docking revealed that Cy7-SE forms five hydrogen bonds with α-BGT, achieving a minimum binding energy of -8. 49 kcal/mol. Visualization of the lowest-energy conformation (**Fig 2**) demonstrates tight binding between Cy7-SE and α-BGT, forming a stable protein-ligand complex at four key residues: Thr-10, Arg-33, Arg-36, and Phe-65. GridBox parameters derived from this docking (**Table 1**) were used for subsequent virtual drug screening.

**Table 1:** GridBox Parameters for Computer-Aided Virtual Drug Screening.

Site	Parameter
center_x	26. 922
center_y	39. 057
center_z	22. 024
size_x	31. 5
size_y	36. 75
size_z	22. 5

### α-BGT-binding molecules identified via batch molecular docking

**Fig 3:**
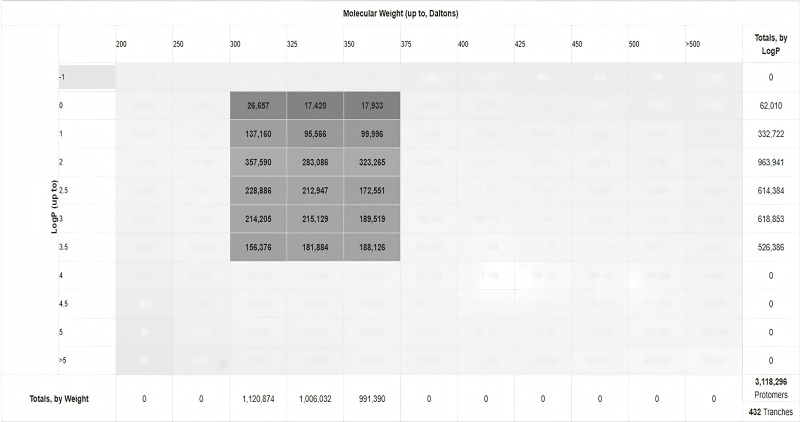
ZINC database screening results. Gray area: Screened molecules; X-axis: logP; Y-axis: Molecular weight; Bottom: Molecular count by MW; Right: Molecular count by logP.

**Fig 4:**
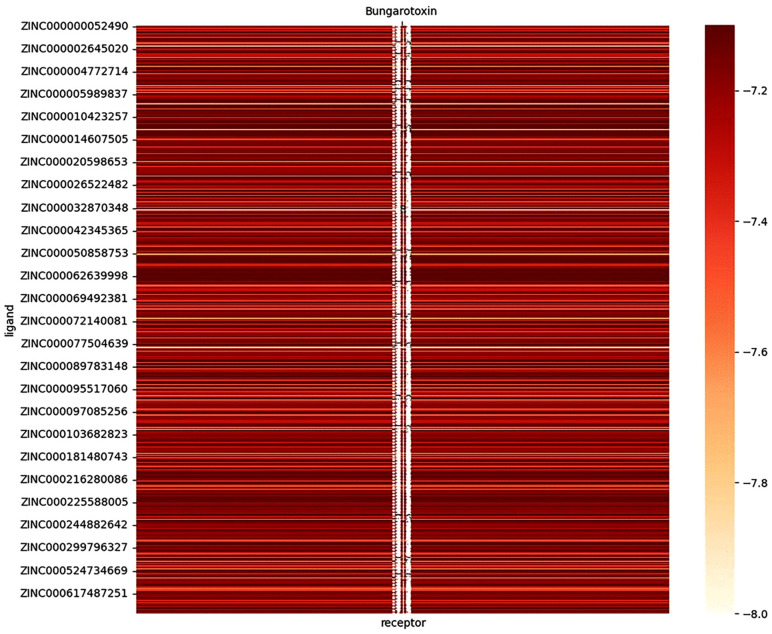
Heatmap of binding energies from batch molecular docking between selected compounds and α-bungarotoxin. Darker hues: Higher binding energy; Lighter hues: Lower binding energy.

Using the Cy7-SE/α-BGT GridBox parameters, batch docking against the ZINC database identified 3, 118, 296 α-BGT-interacting molecules (**Fig 3**). The top 5% of compounds by binding energy (≤ -7 kcal/mol) were retained, yielding 572 candidates. As shown in **Fig 4**, binding energies for these molecules predominantly ranged from -7. 2 to -7. 6 kcal/mol.

### ADMET, lipinski, and pandas-based screening of α-BGT-binding molecules

**Fig 5:**
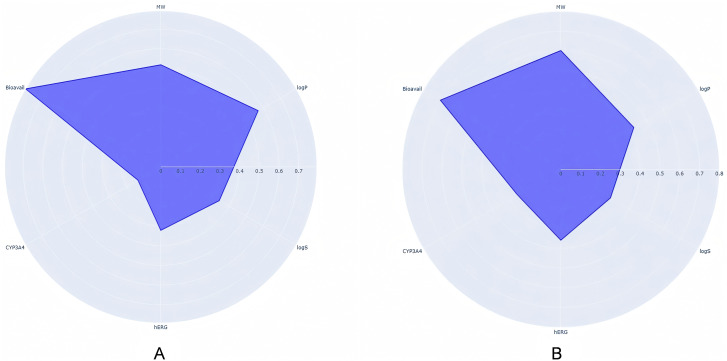
Six-dimensional radar plots of representative screened molecules. A: Lipinski rule-assisted ADMET screening;B: Pandas-weighted screening (P < 0. 05 vs. Lipinski).

ADMET prediction and Lipinski’s rule filtering of the 572 candidates yielded 459 compounds for weighted analysis. Representative six-dimensional radar plots are shown in **Fig 5A**. Pandas-based weighted scoring incorporated bioavailability (Bioavail, weight = 0. 3), hERG inhibition (weight = 0. 2), logS (weight = 0. 2), CYP3A4 inhibition (weight = 0. 2), and molecular weight (MW, weight = 0. 1). The top 20% (102 molecules) were advanced (**Fig 5B**).

**Fig 6:**
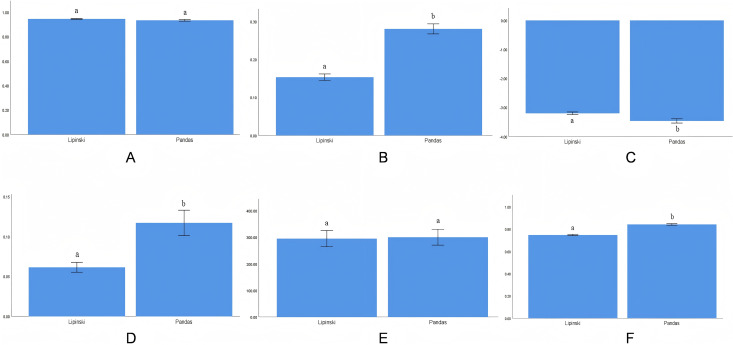
Parameter comparison between screening methods.

As shown in **Fig 6A**, the absence of significant differences in Bioavail parameters between Pandas-weighted screening molecules and Lipinski-filtered molecules (P > 0. 05) results from prior ADMET exclusion of compounds with poor bioavailability. **Fig 6B** demonstrates that Pandas-screened molecules exhibit superior hERG inhibition profiles compared to Lipinski-filtered molecules (P < 0. 05), indicating enhanced cardiac safety. In **Fig 6C**, lower logS values in Pandas-screened molecules (P < 0. 05) reflect reduced aqueous solubility, increased lipophilicity, and improved absorption potential. **Fig 6D** reveals higher CYP3A4 inhibition parameters in Pandas-screened compounds (P < 0. 05), signifying optimized hepatic metabolism. Consistent with **Fig 6A**, **Fig 6E** shows no significant Bioavail difference between screening methods due to preliminary ADMET filtering. Crucially, **Fig 6F** demonstrates statistically significant enhancement in composite druglikeness scores for Pandas-screened molecules versus Lipinski-filtered counterparts (P < 0. 05), confirming greater average druggability and validating screening success.

### PyMOL-screened α-BGT-binding small molecules

**Fig 7:**
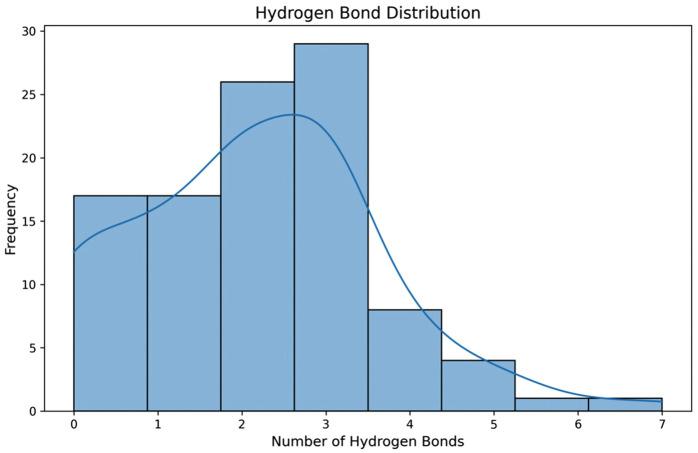
Histogram of hydrogen bond counts between 102 molecules and α-bungarotoxin receptor (PyMOL analysis).

**Fig 8:**
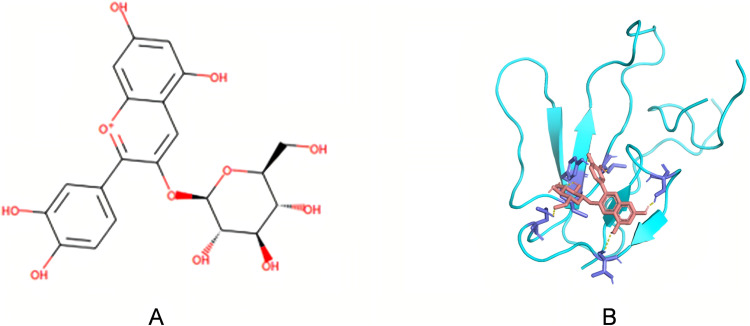
Molecular structure of C3G and its docked conformation with α-bungarotoxin. Fig A displays the molecular structure of C3G, while Fig B visualizes the molecular docking between C3G and α-bungarotoxin. The cyan structure represents the protein, the magenta element denotes the small molecule ligand, yellow lines indicate hydrogen bonds, and blue highlights mark the amino acid residues involved in binding interactions.

The 102 candidate molecules were subjected to batch visualization in PyMOL using their lowest-energy conformations bound to α-neurotoxin. Hydrogen bonding interactions (distance < 3. 3 Å) were quantified, revealing that most compounds formed 2–3 hydrogen bonds with the receptor, with a maximum of 7 bonds observed (**Fig 7**). Through literature-guided analysis, ZINC000034048081 ((2S, 3R, 4S, 5S, 6R)-2- [2-(3, 4-dihydroxyphenyl)-5, 7-dihydroxychromenylium-3-yl]oxy-6-(hydroxymethyl)oxane-3, 4, 5-triol, Cyanidin 3-glucoside, C3G) was selected as a candidate compound to alleviate symptoms of *Bungarus multicinctus* envenomation. The molecular structure of C3G is shown in Fig 8A, with its docked conformation with α-bungarotoxin visualized in **Fig 8B**.

### Molecular dynamics of C3G-α-neurotoxin binding

**Fig 9:**
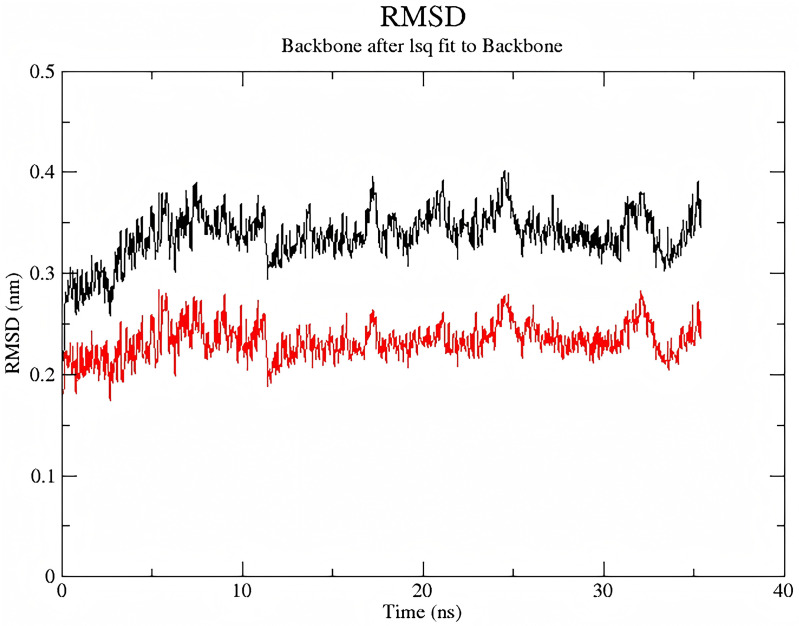
RMSD plot of 35-ns molecular dynamics simulation between C3G and α-bungarotoxin. The black line represents C3G and the red line represents the protein. The RMSD value from their molecular dynamics simulation stabilized below 0. 4 nm after equilibration, indicating stable binding conformation.

Molecular dynamics simulations (**Fig 9**) demonstrated spontaneous binding between C3G and α-bungarotoxin in an aqueous environment, forming a stable complex that validated the virtual screening outcomes.

### Results of surface plasmon resonance analysis for C3G and α-BGT

**Fig 10:**
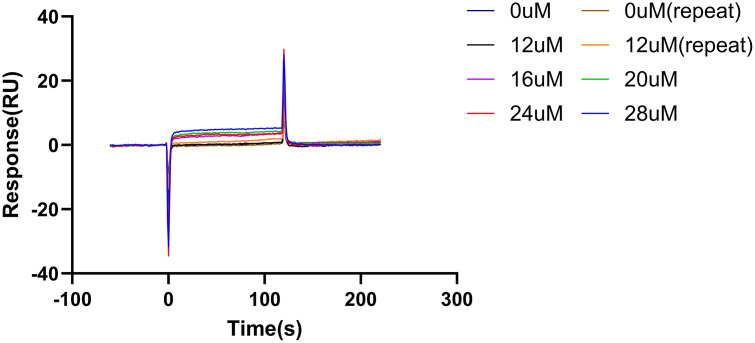
Binding and dissociation curves of α-BGT with C3G.

The results of SPR analysis for the interaction between C3G and α-BGT are shown in Fig 10. At all tested C3G concentration gradients, the sensorgrams displayed a clear binding response trend, where the response value increased over time. This indicates effective molecular binding between C3G and the α-BGT immobilized on the sensor chip surface. Furthermore, the intensity of the binding response showed a distinct concentration dependence, consistent with a typical ligand-analyte binding model. This phenomenon further confirms the existence of direct and saturable binding between C3G and α-BGT. Through fitting analysis of the SPR sensorgram data, this study quantitatively obtained the binding kinetic parameters and affinity constant for the C3G and α-BGT interaction. The system fitting results showed that the association rate constant (ka) was 1. 531 × 10^5^/Ms, and the dissociation rate constant (kd) was 0. 05292/s. Based on these kinetic parameters, the equilibrium dissociation constant (KD) was calculated to be 3. 457 × 10^-7^M, which is in the nanomolar range. This indicates a strong binding capability between C3G and α-BGT. The low chi-square value (Chi² = 3. 05) suggests a high degree of agreement between the experimental data and the theoretical model, confirming the high reliability of the results.

### Effect of C3G mixed with venom and C3G treatment on survival in envenomed mice

**Fig 11:**
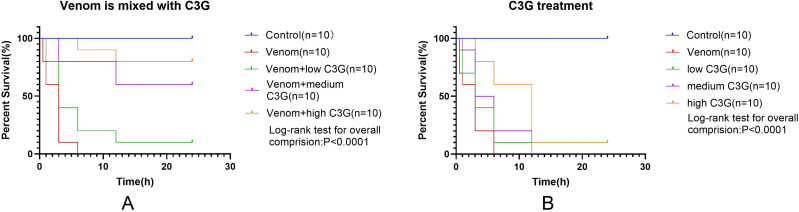
A Effect of*B. multicinctus*venom mixed with varying C3G doses on 24h KM mouse surviva. B Effect of immediate post-envenomation C3G injection on 24h survival.

As shown in **Fig 11A** compared to the control group, the venom group exhibited decreased survival starting at 10 min, with 100% mortality by 6h (P<0.0001). Versus the venom group: The venom+low-dose C3G group showed no significant survival improvement (P > 0. 05); The venom+medium-dose C3G group demonstrated significantly increased 24h survival (60%, P<0.001), with mortality onset delayed from 10 min to 1h; The venom+high-dose C3G group achieved 80% 24h survival (P<0.0001 vs. venom group), with mortality onset delayed to 3h. Both medium and high doses significantly outperformed the low-dose group (P<0.001). In therapeutic intervention studies (immediate post-envenomation C3G injection, Fig 11B): Medium and high-dose groups delayed mortality onset versus venom controls. While low and medium-dose groups reached 100% mortality by 12h, high-dose mice survived beyond this timepoint. At 3h, high-dose survival differed significantly from venom controls (P<0.01). By 6h, only the high-dose group maintained significantly higher survival than venom controls (P<0.001). High-dose mice exhibited longer mean survival time than venom and low-dose groups. No significant survival improvement occurred at 12h/24h across treatment groups, though C3G shows potential for alleviating symptoms of *Bungarus multicinctus* envenomation.

### Impact of C3G on diaphragm and lung histopathology

**Fig 12:**
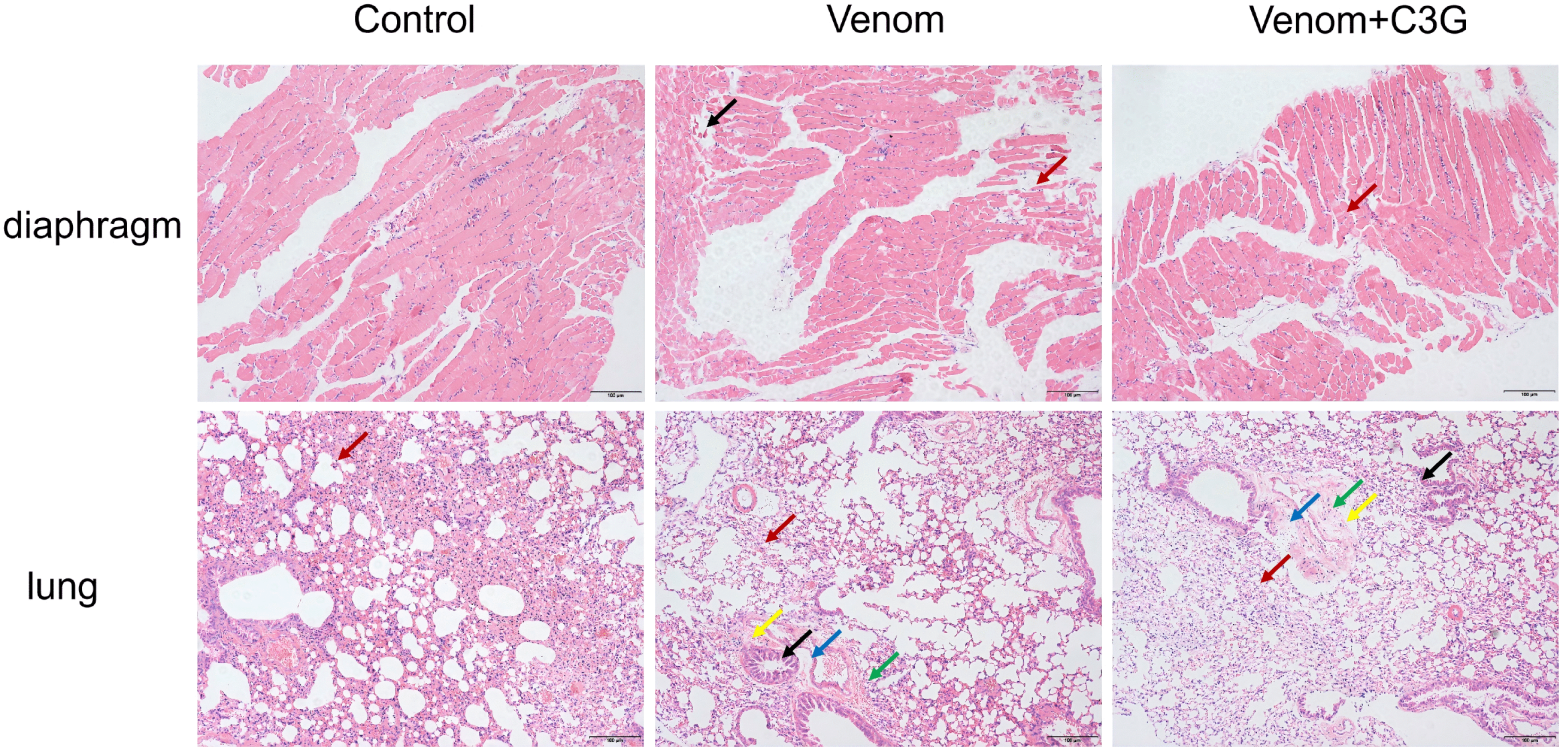
Pathological alterations in pulmonary and diaphragmatic tissues following immediate C3G injection after envenomation(10X).

The results of HE staining for diaphragm and lung tissues are shown in Fig 12. Compared with the normal group, the venom group exhibited necrosis of muscle fibers in the diaphragm (black arrows), along with nuclear pyknosis and fragmentation, eosinophilic staining of the cytoplasm, and occasional lymphocyte infiltration in the interstitium (red arrows). In contrast, the venom + C3G group showed only occasional lymphocyte infiltration in the diaphragm (red arrows) compared to the venom group. Compared with the normal group, the lung tissues of the venom group displayed irregular arrangement of bronchiolar epithelial cells, occasional necrosis and shedding of bronchiolar epithelial cells (black arrows), significant perivascular edema (blue arrows), occasional lymphocyte infiltration (yellow arrows), and extensive perivascular hemorrhage (green arrows). In comparison to the venom group, the venom + C3G group showed occasional lymphocyte infiltration (yellow arrows), occasional necrosis and shedding of bronchiolar epithelial cells (black arrows), mild perivascular edema (blue arrows), and mild perivascular hemorrhage (green arrows) in the lung tissues. All three groups presented mild granulocyte infiltration in the alveolar walls (red arrows). Pathological scoring of diaphragm and lung tissues was performed based on a four‑grade classification system [31], with specific scores provided in Tables 2 and 3.

**Table 2:** Histopathological Scores of Diaphragm Tissues in the Three Groups.

Group	Lesion		
	Necrosis	Bleeding	Inflammatory cell infiltration
Venom	1	0	1
Venom+C3G	0	0	1
Control	0	0	0

**Table 3:** Histopathological Scores of Lung Tissues in the Three Groups.

Group	Lesion			
	Necrosis	Bleeding	Edema	Inflammatory cell infiltration
Venom	1	2	2	1
Venom+C3G	1	1	1	1
Control	0	0	0	1

## Discussion

The venom of *Bungarus multicinctus* contains various neurotoxic components, including α-BGT and β-BGT. The symptoms of envenomation by this snake primarily manifest as neurotoxicity, with studies indicating that the neurotoxin α-bungarotoxin plays a significant role. Cy7-SE, a synthetic fluorophore featuring odd-numbered methylene bridges between nitrogen atoms, forms stable amide bonds with primary amines for protein/antibody labeling [32,33]. Unexpectedly, co-injection of Cy7-SE with *B. multicinctus* venom prevented murine toxicity in vivo, indicating that the venom toxicity is attenuated. However, post-envenomation Cy7-SE administration failed to rescue mice or alleviate symptoms. Molecular docking revealed Cy7-SE binding at Arg-36 of α-bungarotoxin (α-BGT) – a critical residue for α1-nAChR interaction [34]. This binding likely sterically blocks α-BGT’s active site, explaining the mechanism of its toxicity attenuation.

To identify small-molecule compounds capable of alleviating symptoms of *Bungarus multicinctus* envenomation both in vitro and in vivo, we focused on the binding parameters derived from the Cy7-SE/α-bungarotoxin interaction. Leveraging the ZINC database—a comprehensive repository of organic molecules widely used in preliminary virtual drug screening—we screened over 3 million α-bungarotoxin-binding compounds using Cy7-SE-based Grid Box parameters [35]. Autodock Vina [36], a high-throughput molecular docking tool, was employed for batch processing, where lower docking scores indicate stronger binding affinity (≤ -7 kcal/mol signifying robust interactions). Applying these criteria (top 5%, binding energy ≤ -7 kcal/mol) to the ZINC-derived compounds yielded 527 candidates for further evaluation.

ADMET profiling serves as a powerful complementary approach in virtual screening, enabling early exclusion of high-risk compounds and guiding structural optimization to enhance pharmacokinetics [37], Ideal candidates exhibit high bioavailability, favorable lipophilicity/solubility, low enzyme inhibition, and minimal toxicity. Combined with Lipinski’s Rule of Five—which predicts oral bioavailability based on molecular weight, LogP, hydrogen bond donors/acceptors—we established a multidimensional screening framework [38]. Pandas-based weighted analysis of the 527 molecules prioritized 102 compounds with optimal ADMET/Lipinski profiles for subsequent filtering. Hydrogen bonding critically governs binding specificity: compounds forming ≥2 bonds (donor-acceptor distance <3. 3Å) exhibit enhanced toxin-neutralizing potential. From the 102 candidates, we selected 15 molecules meeting these criteria. Literature review identified cyanidin-3-O-glucoside (C3G) for experimental validation—an anthocyanin-derived compound with documented neuroprotective effects. Studies demonstrate C3G mitigates Alzheimer’s and Parkinson’s risks [39], by suppressing neuroinflammation and oxidative stress. Min et al. confirmed its cytoprotection against cerebral ischemia-reperfusion injury [40], while others noted inhibition of NF-κB, caspase-1, and IL-8 expression [41]. C3G also modulates cholinergic function by inhibiting acetylcholinesterase and enhancing ATPase activities [42].

Structurally analogous to Cy7-SE yet endowed with intrinsic antioxidant activity and high α-bungarotoxin affinity, C3G emerged as a prime candidate. Molecular dynamics simulations confirmed spontaneous, stable binding in aqueous environments (validated by RMSD analysis). The surface plasmon resonance assay further demonstrated a strong binding affinity between C3G and α-BGT. To analyze the impact of C3G on the toxic potency of *Bungarus multicinctus* venom, mice were injected with a mixture of C3G and the venom. Within 24 hours, the survival rate of the low-dose C3G group did not show a significant improvement. However, significant increases in survival were observed in both the mid-dose and high-dose groups. Moreover, as the dose of C3G increased, the time to onset of mortality in mice was delayed.

Further investigation into the therapeutic effect of C3G on *Bungarus multicinctus* envenomation was conducted by simulating a scenario of immediate post-bite treatment. Mice were immediately administered C3G following venom injection. The results showed that, compared to the venom-only group, none of the three C3G dose groups exhibited a significantly improved survival rate at 24 hours. However, at the 3-hour and 6-hour time points, the survival rate in the high-dose group was higher than that in the venom-only group. Additionally, the average survival time within 24 hours was prolonged in the C3G-treated groups. These findings indicate that C3G can delay the progression of envenomation symptoms and extend the survival time of mice poisoned by *Bungarus multicinctus* venom.

Histological analysis revealed multi-organ damage in venom-injected mice at 3h, including pulmonary edema, vascular congestion, inflammatory cell infiltration, and muscular edema. In contrast, C3G treatment markedly attenuated venom-induced tissue damage and suppressed inflammatory responses.

C3G demonstrates potential in alleviating the symptoms of *Bungarus multicinctus* envenomation. The broad-spectrum anti-inflammatory and antioxidant properties of C3G provide nonspecific protective effects against secondary damage caused by the venom, improving prognosis by mitigating inflammation and oxidative stress. This overall protective effect, such as prolonging survival time and reducing multi-organ damage, supports the notion that C3G possesses a broader capacity to intervene in the overall pathophysiology induced by the whole venom. However, this does not represent clinically meaningful venom neutralization, thus eliminating the need for comparative testing with antivenom.

In the current field of snake venom research, several small-molecule inhibitors (SMIs) have been proven to exhibit broad-spectrum anti-venom effects. Among them, Varespladib and its prodrug Varespladib-methyl are typical representatives of PLA₂inhibitors. They have been reported to increase survival rates after bites from elapid snakes like *Bungarus multicinctus* and demonstrate in vivo rescue effects post-envenomation [43]. This study found that C3G exhibits activity in delaying the progression of envenomation and reducing tissue damage, suggesting that C3G may represent an auxiliary or alternative strategy distinct from PLA₂inhibitors. C3G primarily functions through antioxidant, anti-inflammatory, and cytoprotective effects [44,45], complementing the mechanism of SMIs like Varespladib, which directly inhibit enzymatic activity. C3G likely improves prognosis by alleviating these secondary injuries.

From a pharmacokinetic perspective, the oral bioavailability of C3G is generally low, and it is rapidly metabolized in vivo with a short half-life. It is primarily metabolized via methylation, glucuronidation, and sulfation into phenolic acid derivatives. Although intravenous injection of C3G in rats leads to its rapid distribution to tissues such as the brain, liver, and kidneys within 15 seconds to 20 minutes, its retention time in tissues and the systemic effects of its metabolites require further investigation [46]. This study employed local injection aiming for C3G to act immediately at the site of venom invasion. However, even at high local doses, the potential toxicity after it enters the systemic circulation needs evaluation. Existing literature mainly reports the benefits of C3G at lower oral doses, and there is no direct data on the safety and toxicity of such high-dose local injection of C3G in humans. Therefore, although this study observed that C3G can delay envenomation symptoms, its clinical safety (e. g., whether it causes local irritation or systemic toxicity such as effects on liver and kidney function) and its metabolic clearance capacity in vivo are critical issues to be addressed in future preclinical and clinical research.

## Conclusion

This study successfully leveraged Cy7-SE’s venom-interacting mechanism to computationally identify and experimentally validate the natural compound C3G as a symptomatic ameliorating candidate for *B. multicinctus* envenomation. C3G delays disease progression and alleviates secondary tissue damage in envenomed mice, thereby improving their survival rate, and acts as an adjuvant supportive option for the clinical management of krait bites.

Currently, the survival benefit conferred by C3G is restricted by its requirement for an extremely high molar excess, a narrow administration window limited to co-injection or immediate post-envenomation intervention, alongside incomplete mechanistic evidence and speculative translational value. These limitations must be explicitly acknowledged. Future research should employ proteomics, toxicology, or functional analyses to evaluate the direct or indirect effects of C3G on other major toxic components in *Bungarus multicinctus* venom (such as PLA₂and neurotoxin isoforms) to construct a more comprehensive explanatory model of its mechanism.

## Supporting information

S1 File Analysis Code for Batch Molecular Docking of α-BGT with Small Molecule Compounds. This code is a batch execution script for Autodock Vina.
(PDF)

S2 File Python-Based Screening of Small Molecules Binding to α-BGT. This Python script screens small molecules ranking in the top 5% of binding energy scores.
(PDF)

S3 File ADMET and Lipinski’s Rule Screening for α-BGT-Binding Small Molecules. This Python script performs ADMET prediction analysis and Lipinski’s rule screening.
(PDF)

S4 File Pandas and PyMOL-Enabled Screening of α-BGT-Targeting Small Molecules. This Python script utilizes Pandas for weighted analysis of results (retaining top 20%), employs PyMOL for batch visualization of ADMET-filtered outputs, extracts the lowest-energy conformation, loads protein structure files to generate images, and calculates/saves hydrogen bond data between ligands and receptors.
(PDF)

S5 File Molecular Dynamics Simulation and Analysis of Cyanidin-3-Glucoside Bound to α-BGT. This code performs molecular dynamics simulations using GROMACS.
(PDF)
