# Supplementary material for: Cyanidin-3-O-glucoside (C3G): A natural small-molecule compound for alleviating envenomation symptoms Induced by Bungarus multicinctus
Source: PLoS Negl Trop Dis. 2026 Apr 7;20(4):e0014207. doi: 10.1371/journal.pntd.0014207 (PMC13155680; doi:10.1371/journal.pntd.0014207)
Supplement: S1 File — This code is a batch execution script for Autodock Vina. (PDF) [file pntd.0014207.s001.pdf]

Script for Batch Molecular Docking Execution Using Autodock Vina:

```
@echo
 for %%f in (*.pdbqt) do (
  echo Processing ligand %%f
  if not exist "%%~nf" mkdir "%%~nf"
  vina --config conf.txt --ligand %%f --out "%%~nf"/out.pdbqt
--log "%%~nf"/log.txt)
Exit
```

Script for Extracting Autodock Vina Batch Docking Results into Binding Energy Datasets:

```
@echo off
> results.txt    REM    Clean or Create    results.txt
for /d %%f in (*) do (
    if exist %%~nf/log.txt (
        for /f "skip=4 tokens=1,2 delims= " %%x in (%%~nf/log.txt) do (
            if %%x==1 (
                echo %%~nf,%%y >> results.txt
            )
        )
    )
)
exit
```
