# Supplementary material for: Cyanidin-3-O-glucoside (C3G): A natural small-molecule compound for alleviating envenomation symptoms Induced by Bungarus multicinctus
Source: PLoS Negl Trop Dis. 2026 Apr 7;20(4):e0014207. doi: 10.1371/journal.pntd.0014207 (PMC13155680; doi:10.1371/journal.pntd.0014207)
Supplement: S2 File — This Python script screens small molecules ranking in the top 5% of binding energy scores. (PDF) [file pntd.0014207.s002.pdf]

```
import pandas as pd
import os
import shutil
# Parameter configuration
excel_path = "Docking Results.xlsx"        # Path to sorted Excel file
pdbqt_dir = "Source Directory Path"          # Directory containing original PDBQT files
output_dir = "top5%_results"     # Output directory for selected files
score_col = "score"                         # Column name for binding energy scores in Excel
name_col = "ligand_name"                 # Column name for ligand identifiers in Excel
# Read and process Excel data
df = pd.read_excel(excel_path)
total = len(df)
top_count = max(1, int(total * 0.05))    # Ensure at least one file is selected
# Extract top 5% ligand names (handle possible file extensions)
top_ligands = df.head(top_count)[name_col].tolist()
top_ligands = {f"{name}_out.pdbqt" for name in top_ligands}      # Generate PDBQT filenames
# Create output directory
os.makedirs(output_dir, exist_ok=True)
# Batch copy selected files
copied_count = 0
for filename in os.listdir(pdbqt_dir):
```

```
        if filename in top_ligands and filename.endswith(".pdbqt"):
            src_path = os.path.join(pdbqt_dir, filename)
            dst_path = os.path.join(output_dir, filename)
            shutil.copy2(src_path, dst_path)    # Preserve file metadata
            copied_count += 1
            print(f"Copied: {filename}")
    print(f"\nComplete! Target files: {len(top_ligands)}, Successfully copied: {copied_count}")
```
