# Supplementary material for: Cyanidin-3-O-glucoside (C3G): A natural small-molecule compound for alleviating envenomation symptoms Induced by Bungarus multicinctus
Source: PLoS Negl Trop Dis. 2026 Apr 7;20(4):e0014207. doi: 10.1371/journal.pntd.0014207 (PMC13155680; doi:10.1371/journal.pntd.0014207)
Supplement: S3 File — This Python script performs ADMET prediction analysis and Lipinski’s rule screening. (PDF) [file pntd.0014207.s003.pdf]

Python Script for ADMET Prediction Analysis of Small Molecules：

```
import os
from rdkit import Chem
from rdkit.Chem import AllChem
from admet_ai import ADMETModel
import pandas as pd
from tqdm import tqdm
# Fix environment encoding issues（required on Windows）
try:
    import locale
    locale.setlocale(locale.LC_ALL, 'en_US.utf-8')
except:
    pass
# Initialize model（compatible with PyTorch 2.5+）
model = ADMETModel()
def mol_to_smiles(mol_path):
"""Convert MOL/SDF file to canonical SMILES"""
   try:
       # Uniform processing of SDF/MOL files
       if mol_path.endswith('.sdf'):
mol=Chem.MolFromMolBlock(open(mol_path).read())
        else:
          mol = Chem.MolFromMolFile(mol_path)
        if mol is None:
          return None
        # Standardization processing
```

```
            mol = Chem.AddHs(mol)
            AllChem.Compute2DCoords(mol)
            return Chem.MolToSmiles(mol, isomericSmiles=True,
canonical=True)
        except:
            return None
 def batch_admet_analysis(input_dir, output_file='admet_results.csv'):
   """Main function for batch ADMET analysis"""
      results = []
      error_files = []
      # Retrieve all molecular files
      mol_files = [f for f in os.listdir(input_dir)
                   if f.endswith(('.mol', '.sdf'))]
      for filename in tqdm(mol_files, desc='Processing Molecules'):
          file_path = os.path.join(input_dir, filename)
          # Convert to SMILES
          smile = mol_to_smiles(file_path)
          if not smile:
              error_files.append(filename)
              continue
           # ADMET prediction
   try:
              pred = model.predict(smile)
              results.append({
                  'File': filename,
                  'SMILES': smile,
```

```
                **pred
            })
        except Exception as e:
            error_files.append(filename)
            print(f"Error in {filename}: {str(e)}")
    # Save results
    df = pd.DataFrame(results)
  df.to_csv(output_file, index=False)
    # Output statistical information
    print(f"\nSuccess: {len(results)} molecules")
    print(f"Failed: {len(error_files)} molecules")
    if error_files:
        print("Problematic files:")
        print('\n'.join(error_files))
    return df
if __name__ == "__main__":
# Input directory containing .mol or .sdf files
   batch_admet_analysis(
input_dir="path/to/your/molecules",
output_file="admet_predictions.csv"
   )
```

}

Python Script for Reading ADMET Results, Applying Lipinski's Rule Screening, and Saving Filtered Outputs to Specified Paths：

```
import pandas as pd
import os
from pathlib import Path
def filter_admet_results(
    input_path="D:\\Vinadock\\ADMET_Results\\admet_prediction.x
lsx",
    output_dir="D:\\Vinadock\\ADMET_Results",
    output_name="admet_filtered.xlsx"
):
    # Ensure output directory exists
    Path(output_dir).mkdir(parents=True, exist_ok=True)
    # Read ADMET results
    df = pd.read_excel(input_path)
    # Column name mapping
    col_map = {
        'Molecular Weight': 'MW',
        'LogP': 'LogP',
        'Water Solubility': 'logS',
        'HERG Inhibition': 'hERG',
        'CYP3A4 Inhibition': 'CYP3A4',
        'Bioavailability': 'Bioavail'
    }
    df = df.rename(columns=col_map)
    # Basic drug screening rules
```

```
    filters = [
        df['MW'] <= 500,                # Molecular weight ≤ 500
        df['LogP'].between(0, 5),        # Lipophilicity 0-5
        df['logS'] >= -4,              # Water solubility ≥ 1e-4 mol/L
        df['hERG'] <= 0.5,              # Weak hERG inhibition
        df['CYP3A4'] <= 0.05,           # Weak CYP3A4 inhibition
        df['Bioavail'] >= 0.5            # Bioavailability ≥ 50%
    ]
    # Apply filtering conditions
    filtered_df = df[pd.concat(filters, axis=1).all(axis=1)]
    # Add drug-likeness score
    filtered_df['Druglikeness'] = (
        0.5 * (filtered_df['MW']/500) +
        0.3 * (filtered_df['LogP']/5) +
        0.2 * (1 - filtered_df['logS']/4)
    ).round(2)
    # Save results
    output_path = os.path.join(output_dir, output_name)
    filtered_df.to_excel(output_path, index=False)
    print(f"Qualified molecules count：{len(filtered_df)}/{len(df)}")
    print(f"Results saved to：{output_path}")
if __name__ == "__main__":
    filter_admet_results()
```
