# Supplementary material for: Cyanidin-3-O-glucoside (C3G): A natural small-molecule compound for alleviating envenomation symptoms Induced by Bungarus multicinctus
Source: PLoS Negl Trop Dis. 2026 Apr 7;20(4):e0014207. doi: 10.1371/journal.pntd.0014207 (PMC13155680; doi:10.1371/journal.pntd.0014207)
Supplement: S4 File — This Python script utilizes Pandas for weighted analysis of results (retaining top 20%), employs PyMOL for batch visualization of ADMET-filtered outputs, extracts the lowest-energy conformation, loads protein structure files to generate images, and calculates/saves hydrogen bond data between ligands and receptors. (PDF) [file pntd.0014207.s004.pdf]

Python Script for Weighted Analysis of Preliminary ADMET Screening Results Using Pandas Library with Top 20% Retention Filter：

```
import pandas as pd
import os
df = pd.read_csv('admet_results.csv')
# Weight assignment (adjust based on project requirements)
df['Comprehensive Score'] = (
    0.3 * df['Bioavail'] +
    0.2 * (1 - df['hERG']) +
    0.2 * df['logS'] +
    0.2 * (1 - df['CYP3A4']) +
    0.1 * df['MW']
)
# Select top 20% candidate molecules
top_candidates = df.nlargest(int(len(df)*0.2), 'Comprehensive Score')
# Create output directory
output_dir = r'D:\Vinadock'
os.makedirs(output_dir, exist_ok=True)
# Output to CSV file
output_path = os.path.join(output_dir, 'top_candidates.csv')
top_candidates.to_csv(output_path,index=False,encoding='utf-8-sig)
print(f'Top 20% candidate molecules have been successfully saved to：{output_path}')
```

Python Script for Batch Visualization Analysis of ADMET-Filtered Molecules Using PyMOL to Extract Hydrogen Bonding Patterns with Bungarus multicinctus Venom Protein：

```
import os
from pymol import cmd
def extract_lowest_energy_model(input_path, output_path):
    """Extract the lowest binding energy conformation"""
        with open(input_path, 'r') as f:
            content = f.read()
        models = []
        current_model = []
        min_energy = float('inf')
        best_model = None
        # Parse PDBQT file
        for line in content.split('\n'):
            if line.startswith('MODEL'):
                if current_model:
                    models.append(current_model)
                current_model = [line]
            elif line.startswith('ENDMDL'):
                current_model.append(line)
                models.append(current_model)
                current_model = []
            else:
                current_model.append(line)
        # Find conformation with lowest energy
```

```
        for model in models:
            for line in model:
                if line.startswith('REMARK VINA RESULT'):
                    energy = float(line.split()[3])
                    if energy < min_energy:
                        min_energy = energy
                        best_model = model
                    break
        # Write to temporary file
        if best_model:
            with open(output_path, 'w') as f:
                f.write('\n'.join(best_model))
# Path settings
ligands_dir = r'D:\Vinadock\Docking_Results\ligands'
protein_path = r'D:\Vinadock\Docking_Results\ctx.pdb'
output_dir = r'D:\Vinadock\Docking_Results\visual'
# Create output directory
os.makedirs(output_dir, exist_ok=True)
# Load protein structure
cmd.load(protein_path, 'protein')
cmd.hide('everything', 'protein')
cmd.show('cartoon', 'protein')
cmd.color('gray', 'protein')
# Iterate through docking results
for filename in os.listdir(ligands_dir):
        if filename.endswith('.pdbqt'):
```

```
        ligand_id = filename.split('.')[0]    # Correction: Get ligand ID
        input_path = os.path.join(ligands_dir, filename)
        temp_path = os.path.join(output_dir, 'temp.pdbqt')
        # Extract lowest energy conformation
        extract_lowest_energy_model(input_path, temp_path)
        # Load ligand
        cmd.load(temp_path, 'ligand')
        os.remove(temp_path)    # Delete temporary file
        # Set display style
        cmd.show('sticks', 'ligand')
        cmd.color('magenta', 'ligand')
        cmd.orient('ligand')
        # Calculate hydrogen bonds (distance < 3.3Å)
        hbonds = cmd.find_pairs('protein and (name N or name O)',
    'ligand and (name N or name O)',
                                mode=1,    # 1=polar atoms only
                                cutoff=3.3)
        # Create hydrogen bond visualization
        cmd.delete('hbonds')
        cmd.distance('hbonds', 'protein', 'ligand',
                     cutoff=3.3, mode=2)
        cmd.set('dash_gap', 0.5)
        cmd.set('dash_color', 'red')
        # Save results
        output_prefix = os.path.join(output_dir, ligand_id)
```

```
        # Save image
        cmd.png(f"{output_prefix}.png", width=800, height=600, dpi=300)
        # Save hydrogen bond information
        with open(f"{output_prefix}_hbonds.txt", 'w') as f:
            f.write(f"Total hydrogen bonds: {len(hbonds)}\n")
            for bond in hbonds:
                f.write(f"Protein atom {bond[0]} - Ligand atom {bond[1]}\n")  # Correction: Output atom info
        # Clean up the scene
        cmd.delete('ligand')
        cmd.delete('hbonds')
    cmd.quit()
```
