# Supplementary material for: Cyanidin-3-O-glucoside (C3G): A natural small-molecule compound for alleviating envenomation symptoms Induced by Bungarus multicinctus
Source: PLoS Negl Trop Dis. 2026 Apr 7;20(4):e0014207. doi: 10.1371/journal.pntd.0014207 (PMC13155680; doi:10.1371/journal.pntd.0014207)
Supplement: S5 File — This code performs molecular dynamics simulations using GROMACS. (PDF) [file pntd.0014207.s005.pdf]

Generate topology and coordinate files

gmx pdb2gmx -f AAAA.pdb -o AAAA_processed.gro -p topol.top -ignh

Select force field corresponding to the ligand-receptor system

Choose spc (GROMACS classical small molecule force field)

Add simulation box (minimum boundary distance typically ⩾0.85 nm)

gmx editconf -f AAAA_processed.gro -o AAAA_newbox.gro -c -d 1.0 -bt cubic

Add water model

gmxsolvate -cp AAAA_newbox.gro -cs spc216.gro -o AAAA_solv.gro -p topol.top

Add ions

gmx grompp -f ions.mdp -c AAAA_solv.gro -p topol.top -o ions.tpr–maxwarn 1

Energy minimization

(1) Steepest descent method

gmx grompp -f em1.mdp -c solv_ions.gro -p topol.top -o em.tpr–maxwarn 7

gmx mdrun -v -deffnm em

(2) Conjugate gradient method

gmx grompp -f em2.mdp -c em.gro -p topol.top -o em.tpr–maxwarn 7

gmx mdrun -v -deffnm em

Conformational restraints

gmx genrestr -f BBB.gro -o posre_BBB.itp -fc 1000 1000 1000

2

gmx make_ndx -f em.gro -o index.ndx

1 | 13

q

NVT equilibration

gmx grompp -f nvt.mdp -c em.gro -r em.gro -p topol.top -n index.ndx -o nvt.tpr–maxwarn 6

gmx mdrun -v -deffnm nvt

NPT equilibration

gmx grompp -f npt.mdp -c nvt.gro -r nvt.gro -t nvt.cpt -p topol.top –n index.ndx -o npt.tpr–maxwarn 6

gmx mdrun –v -deffnm npt

Run production MD simulation

gmx grompp -f md.mdp -c npt.gro -r npt.gro -t npt.cpt -p topol.top –n index.ndx -o md_0_1.tpr–maxwarn 6

gmx mdrun -v -deffnm md_0_1
